# Beyond the Skin: Exploring the Gut–Skin Axis in Chronic Spontaneous Urticaria and Other Inflammatory Skin Diseases

**DOI:** 10.3390/biomedicines13082014

**Published:** 2025-08-19

**Authors:** Laura Haidar, Camelia Felicia Bănărescu, Cristina Uța, Elena-Larisa Zimbru, Răzvan-Ionuț Zimbru, Alexandru Tîrziu, Raul Pătrașcu, Alina-Florina Șerb, Marius Georgescu, Daciana Nistor, Carmen Panaitescu

**Affiliations:** 1 Center of Immuno-Physiology and Biotechnologies, Department of Functional Sciences, “Victor Babeș” University of Medicine and Pharmacy, 2 Eftimie Murgu Square, 300041 Timisoara, Romania; 2 Timis County Emergency Clinical Hospital “Pius Brinzeu”, 156 Liviu Rebreanu Bd., 300723 Timisoara, Romania; 3 Multidisciplinary Heart Research Center, “Victor Babeș” University of Medicine and Pharmacy, 300041 Timisoara, Romania; 4 Cardiovascular Disease Institute Timisoara, Gheorghe Adam Street, No. 13A, 300310 Timisoara, Romania; 5 Department of Biochemistry and Pharmacology, Biochemistry Discipline, “Victor Babeș” University of Medicine and Pharmacy Timișoara, 2 Eftimie Murgu Square, 300041 Timisoara, Romania

**Keywords:** chronic spontaneous urticaria (CSU), gut microbiota, gut–skin axis, dysbiosis, mast cells, probiotics, personalized medicine

## Abstract

Emerging evidence suggests a critical role of the gut microbiome in modulating systemic immune responses, with increasing relevance in dermatological diseases. Chronic spontaneous urticaria (CSU), traditionally viewed as an isolated cutaneous disorder, is now recognized as a systemic immune condition involving complex interactions between innate and adaptive immunity, mast cell dysregulation, and non-IgE-mediated pathways. This review explores the gut–skin axis as a unifying concept linking intestinal dysbiosis to inflammatory skin diseases, including atopic dermatitis, psoriasis, rosacea, and acne. Special emphasis is placed on CSU, where altered gut microbial composition, characterized by reduced diversity, depletion of short-chain fatty acid-producing bacteria, and expansion of Proteobacteria, may contribute to increased intestinal permeability, systemic immune activation via toll-like receptors, and heightened mast cell sensitivity. We discuss findings from animal models demonstrating that gut microbiota modulation can attenuate mast cell hyperreactivity and reduce urticarial symptoms. In parallel, we examine clinical evidence supporting the potential role of probiotics, prebiotics, dietary interventions, and fecal microbiota transplantation as adjunctive strategies in CSU management. Despite promising findings, challenges remain in translating microbiome research into effective therapies due to interindividual variability, the complexity of host–microbiome interactions, and a lack of standardized protocols. Future research should focus on identifying predictive microbial patterns and developing personalized microbiome-targeted interventions. Understanding the bidirectional gut–skin relationship may open new therapeutic avenues beyond symptomatic treatment, positioning the microbiome as a novel target in CSU and related inflammatory dermatoses.

## 1. Introduction

Urticaria is a common dermatological condition characterized by the sudden appearance of wheals (hives), angioedema, or both [1]. Wheals are transient, edematous, erythematous plaques that are typically associated with intense pruritus. By definition, individual wheals usually resolve within 24 h without residual marks [2]. Angioedema, on the other hand, represents deeper swelling involving the dermis and subcutaneous tissues, often affecting the lips, eyelids, and extremities, and is characterized by sensations of tingling, burning, tightness, or pain rather than itch, with a typically slower resolution that may take up to 72 h [1].

Clinically, urticaria is classified into acute urticaria (symptoms lasting less than six weeks) and chronic urticaria (symptoms for six weeks or longer) [1,3]. Chronic urticaria itself can be further subdivided into chronic spontaneous urticaria (CSU) and chronic inducible urticaria (CIndU). In CSU, wheals and/or angioedema occur spontaneously without an identifiable external trigger, whereas in CIndU, symptoms are reproducibly induced by specific stimuli such as cold, pressure, heat, or exercise [1,3,4].

CSU accounts for the majority of chronic urticaria cases; prevalence tends to be slightly higher in women and appears to vary across populations, being more common in Asian populations compared to European and American populations [5]. CSU significantly impairs quality of life, causing sleep disturbances, emotional distress, and social withdrawal [6,7,8]. Although often self-limiting, CSU can persist for years in a substantial subset of patients [6,9].

Current therapeutic approaches aim to control symptoms through stepwise escalation, starting with second-generation antihistamines and progressing to biologic therapies, notably omalizumab, an anti-immunoglobulin E (IgE) monoclonal antibody, or immunosuppressants like cyclosporine in refractory cases [1].

Despite advances in the understanding and management of CSU, its triggers remain elusive in many patients, and systemic factors such as infections, autoimmunity, and, increasingly, alterations in the gut microbiome are being explored as contributors to disease pathogenesis and persistence. These emerging insights open new avenues for research into the gut–skin–immune axis and potential microbiome-targeted therapies in chronic urticaria.

## 2. Systemic Immune Regulation Beyond Classical IgE-Mediated Hypersensitivity and the Microbiome

The pathogenesis of CSU is complex and multifactorial. Traditionally, it has been attributed to classical IgE-mediated hypersensitivity reactions and aberrant activation and degranulation of skin mast cells, leading to the release of histamine and other pro-inflammatory mediators [1].

However, increasing evidence suggests that systemic immune dysregulation in CSU extends well beyond the traditional IgE pathway. Recent research has expanded this model, suggesting a broader dysregulation of innate and adaptive immune pathways, involving basophils, eosinophils, T cells, and components of the coagulation system [10,11,12]. Many patients with CSU have no identifiable allergen triggers, and allergen-specific IgE is often absent or irrelevant in their clinical course [13]. Instead, alternative mechanisms, particularly autoimmune and autoallergic processes, have been implicated [14]. In autoimmune CSU, autoantibodies of the IgG class target either FcεRI or IgE itself, directly activating mast cells and basophils in an allergen-independent manner (type IIb autoimmunity) [1,15,16]. In autoallergic CSU, IgE antibodies against self-antigens (such as thyroglobulin or double-stranded DNA) lead to chronic mast cell activation (type I autoimmunity) [9,16].

Moreover, additional layers of immune dysregulation have been identified, involving innate immune cells (e.g., basophils, eosinophils), T cell imbalances including reduced regulatory T cell (Treg) function and enhanced T helper (Th) 2 and Th17 responses [12,17], and activation of the complement and coagulation systems [18,19,20], which further amplify mast cell reactivity. Cytokines such as interleukin (IL)-6, IL-17, and tumor necrosis factor (TNF), rather than histamine alone, have been found elevated in CSU patients, suggesting a broader systemic inflammatory state [12,17].

These insights have fundamentally shifted the understanding of CSU from a local, mast-cell-centric disorder to a complex systemic immune dysregulation, involving both adaptive and innate immune networks. This evolving view has significant therapeutic implications driving the exploration of novel targets beyond antihistamines and anti-IgE strategies, including biologics that modulate broader immune pathways.

Given this expanded understanding of CSU as a systemic immune dysregulation rather than a purely local hypersensitivity reaction, attention has increasingly turned to systemic modulators of immune balance. One of the most intriguing areas of investigation is the role of the gut microbiome, the vast and dynamic community of microorganisms residing within the gastrointestinal tract, in regulating both innate and adaptive immune responses. Disruptions in the gut microbiome composition, known as dysbiosis, have been implicated in a variety of inflammatory and autoimmune conditions, including atopic dermatitis, psoriasis, and systemic lupus erythematosus [21,22,23]. In CSU, alterations in gut microbial composition and diversity, characterized by an imbalance of beneficial and pathogenic bacteria, have been increasingly recognized. These dysbiotic states can lead to increased gut permeability, allowing microbial products and metabolites to enter the systemic circulation, acting as biotic stressors [24] and potentially priming immune cells and contributing to chronic inflammation characteristic of CSU.

Furthermore, the gut microbiome produces a vast array of metabolites, including short-chain fatty acids (SCFAs), which exert significant immunomodulatory effects. Changes in the production of these metabolites in dysbiosis may influence mast cell activation, basophil function, and the overall immune tolerance within the host, thereby impacting the severity and persistence of CSU symptoms. Exploring the gut–skin axis and examining specific microbial signatures in CSU patients can reveal their functional consequences. This understanding offers a promising path to uncover novel disease mechanisms and identify new therapeutic targets, potentially leading to microbiome-focused interventions that go beyond current drug treatments.

## 3. The Gut–Skin Axis in Inflammatory Skin Diseases

The concept of a gut–skin axis refers to the bidirectional communication between the gastrointestinal microbiota and the skin’s immune and barrier systems [25,26]. Through the production of microbial metabolites, modulation of systemic cytokine levels, and regulation of immune cell differentiation, the gut microbiome exerts a profound influence on systemic immune homeostasis [27,28,29].

In healthy individuals, the gut microbiome is a complex and dynamic ecosystem predominantly composed of bacteria from the Firmicutes and Bacteroidetes phyla, with additional contributions from Actinobacteria, Proteobacteria, Verrucomicrobia, and others. This diverse microbial community includes beneficial genera such as *Faecalibacterium*, *Bacteroides*, *Bifidobacterium*, *Lactobacillus*, *Akkermansia*, and *Ruminococcus*, many of which are involved in the fermentation of dietary fibers into SCFAs, modulation of immune tolerance, maintenance of epithelial integrity, and inhibition of pathogenic colonization [30,31]. Notably, species such as *Faecalibacterium prausnitzii*, *Akkermansia muciniphila*, and *Bifidobacterium longum* have been associated with anti-inflammatory properties and metabolic regulation, underscoring their role in maintaining intestinal and systemic immune balance [32,33]. The major bacterial phyla, along with representative genera and notable species that are commonly part of the normal (eubiotic) human gut microbiome, are listed in Table 1.

A stable and diverse gut microbiota is considered a hallmark of health, contributing to mucosal barrier function, immunological education, and resistance to perturbations. However, when this equilibrium is disrupted, a state referred to as dysbiosis, it can lead to increased intestinal permeability, aberrant immune activation, and systemic inflammation.

Dysbiosis, characterized by reduced microbial diversity, loss of beneficial commensals, and overgrowth of potentially pro-inflammatory species, has been linked to a range of chronic skin inflammatory conditions, including atopic dermatitis [37,38], psoriasis [26,39], rosacea [40,41], and acne [42,43]. In these disorders, alterations in gut microbiota composition have been associated with impaired epithelial barrier function, systemic low-grade inflammation, and a shift toward Th2 or Th17-skewed immune responses [44]. While these conditions share a common foundation of dysbiosis, each is characterized by distinctive microbial signatures and immune pathways that contribute to their specific pathologies. Given that CSU shares similar immunological features, including mast cell hyperreactivity, Th2/Th17 polarization, and elevated systemic inflammatory markers, it is plausible that disruptions in the gut microbiome may contribute to the pathogenesis and persistence of urticaria. Although research in this area remains at an early stage, preliminary studies suggest a potential role for the gut microbiota in influencing disease severity, duration, and therapeutic responsiveness in CSU.

The gut microbiome exerts widespread influence on host immunity, not only through its taxonomic composition but also via its metabolic and structural impact on the intestinal environment. One central concept is the phenomenon of increased intestinal permeability, commonly referred to as “leaky gut,” in which the integrity of the epithelial barrier is compromised. This allows translocation of microbial products such as lipopolysaccharides (LPS), peptidoglycans, and other pathogen-associated molecular patterns (PAMPs) into systemic circulation [45]. These molecules are recognized by pattern recognition receptors (PRRs) on immune cells, especially toll-like receptors (TLRs), leading to activation of downstream signaling cascades and the production of pro-inflammatory cytokines including IL-1β, IL-6, IL-17, TNF, and C-reactive protein (CRP). Therefore, the presence of these pro-inflammatory molecules can trigger low-grade systemic inflammation, activate innate immune cells, and potentially prime mast cells or basophils, all of which may contribute to disease flares in predisposed individuals [46,47].

This systemic low-grade inflammation not only sustains immune activation but may also directly affect mast cell biology. Pro-inflammatory cytokines have been shown to increase mast cell receptor expression (e.g., FcεRI), enhance mast cell survival and tissue recruitment, and lower the threshold for degranulation in response to both immunologic and non-immunologic triggers [48,49,50,51]. Moreover, LPS can directly activate mast cells through TLR4 in the absence of antigen-specific IgE [52], particularly in tissues rich in mucosal mast cells, such as the skin and gut. This “primed” state of mast cells in a dysregulated systemic environment could contribute to their spontaneous or exaggerated degranulation, even in the absence of known allergens, offering a plausible explanation for the persistent, idiopathic flares in CSU.

Additionally, microbial metabolites, particularly SCFAs such as butyrate, propionate, and acetate, play an essential role in immune homeostasis [53,54]. SCFAs are produced by fermentation of dietary fibers by commensal bacteria (e.g., *Lactobacillus* spp., *Bifidobacterium* spp., *Roseburia* spp., *Faecalibacterium prausnitzii*) [55,56] and are known to support intestinal barrier integrity, promote regulatory T cell (Treg) differentiation, and suppress pro-inflammatory Th17 responses [57,58]. Disruption of SCFA-producing microbiota may therefore tip the balance toward a pro-inflammatory Th17-skewed immune profile, which has been implicated in various inflammatory skin diseases and may plausibly influence the course of chronic urticaria.

**Figure 1:**
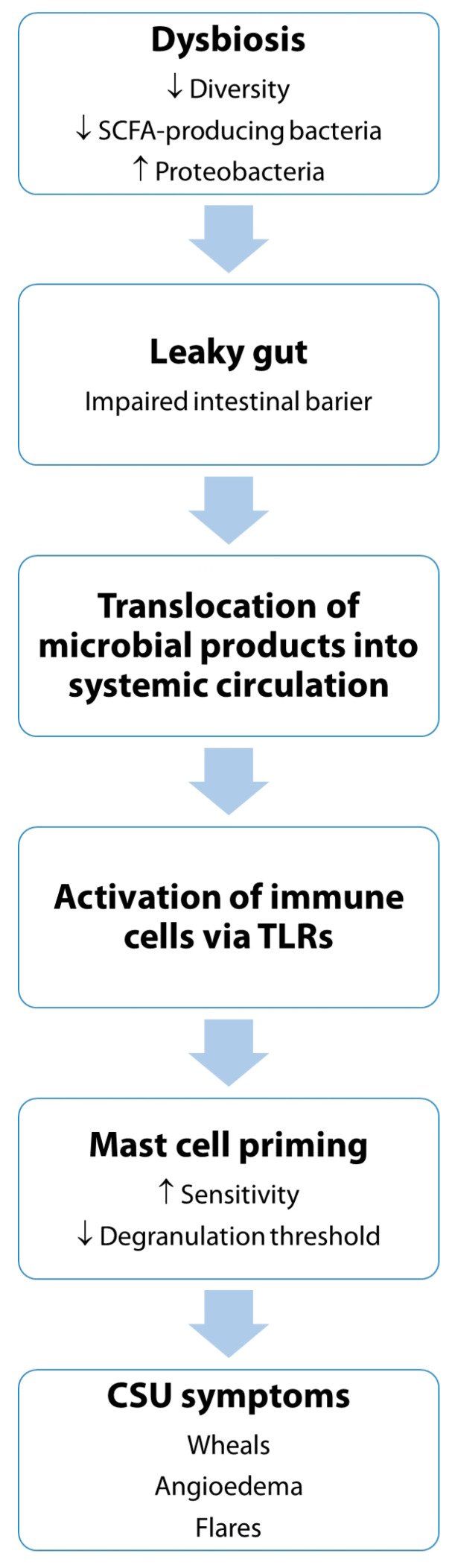
Proposed mechanistic pathway linking gut microbiota dysbiosis to CSU. Gut dysbiosis, characterized by reduced microbial diversity, a decrease in SCFA-producing bacteria, and an overrepresentation of *Proteobacteria*, contributes to the disruption of intestinal barrier integrity, a phenomenon commonly referred to as “leaky gut.” This impaired barrier function allows translocation of microbial products, such as LPS, into the systemic circulation. These microbial components can engage TLRs on immune cells, triggering an innate immune response and promoting systemic inflammation. Subsequent activation and priming of mast cells, central effector cells in CSU, results in increased sensitivity and a lower threshold for degranulation. This enhanced reactivity contributes to the release of histamine and other pro-inflammatory mediators, ultimately leading to the clinical symptoms of CSU, including wheals, angioedema, and spontaneous flares. The figure underscores the potential role of the gut–skin axis in modulating disease activity in CSU and highlights the gut microbiome as a possible therapeutic target.

Figure 1 illustrates the proposed mechanistic pathway linking gut dysbiosis to the pathogenesis of CSU.

Collectively, these mechanisms—leaky gut, dysbiosis-driven loss of SCFAs, and the resulting immune imbalance—represent key pathways through which the gut microbiota may contribute to systemic immune activation and peripheral organ involvement, including the skin.

### 3.1. Atopic Dermatitis and Gut Dysbiosis

In atopic dermatitis (AD), several studies have consistently demonstrated a link between gut microbiota imbalance and disease severity [59,60]. Infants who later develop AD often show a lower abundance of beneficial bacteria, such as *Bifidobacterium* and *Lactobacillus*, in their early microbiota profiles compared to healthy controls [61,62]. This is often more pronounced in AD, especially in infants, compared to conditions like psoriasis, where different beneficial species might be affected [63], and suggests that early-life microbial colonization plays a role in immune system training and tolerance development.

Moreover, gut dysbiosis in AD is associated with increased intestinal permeability, leading to translocation of microbial products like LPS into systemic circulation [64]. This systemic immune activation enhances Th2 skewing, promoting elevated IgE production, eosinophilia, and impaired skin barrier function via reduced expression of filaggrin and other epidermal proteins, hallmarks of AD pathogenesis that distinguish it from other inflammatory skin diseases [65,66].

Interventional studies with probiotics (e.g., *Lactobacillus rhamnosus*) have shown a modest reduction in the incidence and severity of AD in some pediatric populations [67,68], suggesting that modulation of the gut microbiota could be a viable preventive or adjunctive therapeutic strategy. However, results remain heterogenous due to differences in probiotic strains, timing, and dosing.

### 3.2. Psoriasis and Gut Dysbiosis

Psoriasis is increasingly viewed as a systemic inflammatory disease, not limited to the skin [69]. Patients with psoriasis have a significantly altered gut microbiota, characterized by reduced bacterial diversity and specific shifts in microbial composition [70]. More specifically, decreased abundance of anti-inflammatory SCFA-producing bacteria (*Faecalibacterium prausnitzii*, *Bacteroides fragilis*) and increased presence of potentially pro-inflammatory Firmicutes (*Ruminococcus gnavus*) and Proteobacteria (*Escherichia coli*) species [70,71].

The loss of SCFA-producing bacteria such as *Faecalibacterium prausnitzii*, which is a key anti-inflammatory species, may lead to enhanced Th17 polarization, which is a hallmark of psoriasis pathogenesis and distinct from the Th2 response in AD [63], thereby promoting systemic and skin inflammation [72].

Furthermore, increased intestinal permeability has been demonstrated in psoriatic patients [39], suggesting that microbial metabolites and endotoxins may enter circulation and perpetuate the inflammatory cascade.

Emerging pilot studies have even explored fecal microbiota transplantation (FMT) as a therapeutic option for psoriasis, though data remain preliminary [73,74].

### 3.3. Rosacea and Gut Dysbiosis

Rosacea, a chronic inflammatory skin condition, is increasingly recognized for its potential systemic connections, including gastrointestinal comorbidities and systemic inflammatory markers [75]. Patients with rosacea often exhibit a significantly altered gut microbiota composition compared to healthy individuals, characterized by reduced diversity and specific shifts in bacterial populations [76].

More specifically, studies have reported an increased abundance of certain potentially pro-inflammatory bacteria, such as *Helicobacter pylori*, though its direct causal role in rosacea remains debated [77]. This increased abundance of *Helicobacter pylori* is a notable finding in rosacea, distinguishing it from other inflammatory skin diseases and suggesting a unique link between this bacterium and the neurovascular changes observed in the condition [78]. Other findings include altered ratios of Firmicutes to Bacteroidetes and changes in the abundance of genera like *Lactobacillus* and *Bifidobacterium*, suggesting dysbiosis that can influence systemic immunity [76,79]. This microbial imbalance may lead to impaired production of beneficial SCFAs, contributing to a pro-inflammatory environment.

Furthermore, evidence suggests that increased intestinal permeability may be present in rosacea patients. This compromised gut barrier can facilitate the translocation of microbial products, such as LPS and other PAMPs, into the systemic circulation. These systemic endotoxins can then perpetuate a low-grade inflammatory cascade, contributing to the neuro-cutaneous inflammation characteristic of rosacea [41,77].

While research is still evolving, pilot studies and anecdotal evidence have explored the potential of gut-modulating therapies, such as probiotics or specific dietary interventions, in managing rosacea symptoms by addressing underlying gut dysbiosis [40,77].

### 3.4. Acne and Gut Dysbiosis

In acne vulgaris, the primary pathological focus has historically been on the skin microbiome (*Cutibacterium acnes* overgrowth) [80,81]. However, emerging evidence suggests that gut dysbiosis may contribute to the systemic inflammatory environment that exacerbates acne.

Studies have shown that individuals with moderate-to-severe acne exhibit a reduced diversity of gut microbiota, an increased Firmicutes-to-Bacteroidetes ratio, a dysbiotic signature often linked to metabolic and inflammatory disorders [82], as well as elevated levels of pro-inflammatory cytokines, possibly linked to gut-derived endotoxemia [42,83].

Additionally, dietary patterns that disrupt gut microbial composition, such as high glycemic load diets, dairy consumption, and low-fiber intake, correlate strongly with acne severity [84]. High-glycemic foods may promote insulin resistance, hyperandrogenism, and increased sebum production, but also directly alter the gut microbiota, more specifically by altering the Firmicutes-to-Bacteroidetes ratio, amplifying systemic inflammation, a key mechanism through which gut changes exacerbate acne vulgaris [85,86]. This pathway highlights a distinct dietary and microbial connection compared to other inflammatory skin conditions.

**Figure 2:**
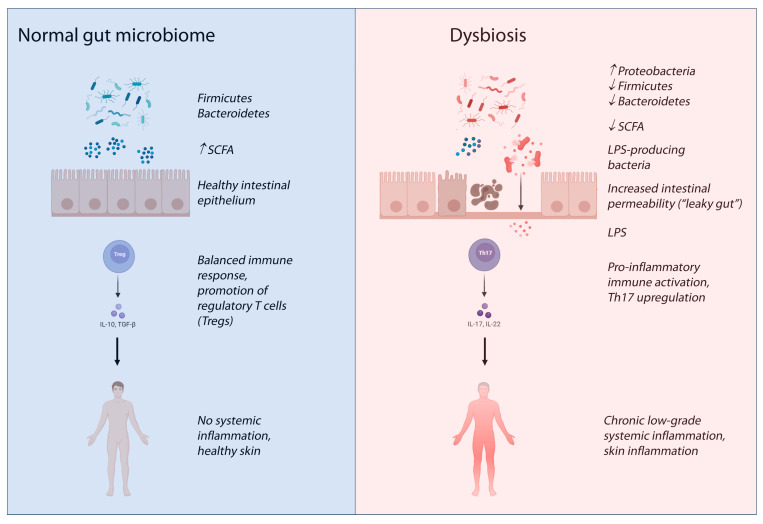
Proposed mechanisms linking gut dysbiosis to inflammatory skin conditions and systemic inflammation. Disruption of the intestinal microbiota can lead to reduced production of short-chain fatty acids (SCFAs), impaired regulatory T cell (Treg) activity, and increased Th17-mediated inflammation. In parallel, altered intestinal permeability (“leaky gut”) allows translocation of microbial components such as lipopolysaccharides (LPS) into systemic circulation, promoting low-grade inflammation and immune cell activation. These processes may contribute to the pathogenesis and exacerbation of inflammatory skin conditions. This image was created by the authors with BioRender.com (accessed on 21 July 2025).

There is preliminary interest in using probiotics to modulate gut microbiota and reduce acne severity, though randomized controlled trials are still scarce [42]. Table 2 provides a comparative overview of the gut microbial changes associated with major inflammatory skin diseases, highlighting common patterns of dysbiosis, impaired barrier function, and systemic inflammation that may also be relevant to chronic spontaneous urticaria, while also displaying the specific dysbiotic patterns and immune shifts that define each condition. Figure 2 shows a proposed mechanism linking gut dysbiosis to inflammatory skin conditions and systemic inflammation.

## 4. Current Evidence Linking the Microbiome to Chronic Spontaneous Urticaria

Given the consistent patterns of gut dysbiosis observed in atopic dermatitis, psoriasis, rosacea, and acne (i.e., reduced microbial diversity, loss of key anti-inflammatory taxa, and increased intestinal permeability), it is plausible that CSU may share similar underlying mechanisms. Like these conditions, CSU is characterized by systemic immune dysregulation, involving mast cell activation, Th2 and Th17 skewing, and low-grade inflammation [12,88]. Although traditionally viewed as a mast cell-driven disease triggered by IgE or autoantibodies, emerging perspectives position CSU within a broader framework of immune system imbalance. As such, disturbances in the gut microbiota could theoretically contribute to mast cell priming, loss of tolerance, or enhanced reactivity through systemic immune modulation. Preliminary findings of altered gut microbiota composition in CSU patients [89,90] further support this hypothesis, opening the door to exploring the gut–skin–immune axis as a potential contributor to urticaria pathogenesis. The potential relationship between gut microbiota and CSU is a relatively new area of investigation, yet it is increasingly drawing attention due to parallels with other inflammatory skin diseases and the systemic nature of CSU [91]. Although data remain limited and largely exploratory, a growing number of studies suggest that gut microbial dysbiosis may play a role in the development or persistence of CSU [92,93], particularly by influencing systemic immune tone, mast cell activation thresholds, and mucosal immune balance [94,95].

One of the earliest findings replicated across several small observational studies is the reduced alpha diversity (diversity within a single sample or a single individual’s microbial community) of gut microbiota in CSU patients [89,96]. Lower microbial diversity has been associated with decreased resilience of the gut ecosystem and a higher propensity toward inflammatory responses [97]. In a 2020 study by Wang et al., gut microbiota analysis in CSU patients showed significantly lower microbial richness and evenness compared to healthy controls, particularly a reduction in species belonging to the Firmicutes phylum, which includes multiple SCFA-producing genera [96]. These beneficial bacteria are thought to support intestinal epithelial integrity, modulate Treg activity, and contribute to immune tolerance. However, other studies reported beta-diversity differences without significant reduction in alpha-diversity but still noted distinct clustering corresponding to CSU status [98].

Additionally, several studies have identified specific shifts in microbial composition in CSU patients, though precise bacterial profiles can vary depending on geographical location, dietary habits, and methodology [90,99,100]. A reduction in *Bifidobacterium* and *Faecalibacterium prausnitzii*, two genera frequently associated with anti-inflammatory functions, has been reported [92]. *Faecalibacterium*, in particular, is known to produce butyrate, an SCFA that promotes Treg differentiation and suppresses Th17-mediated inflammation [101]. The loss of butyrate-producing taxa may thus impair the host’s ability to regulate peripheral inflammation, contributing to a heightened inflammatory baseline conducive to mast cell activation. Similarly, species from the *Bifidobacterium* and *Lactobacillus* genera, recognized for their immunomodulatory and barrier-strengthening properties, may also be diminished [90,102].

On the other hand, several studies have noted an increase in members of the *Proteobacteria* phylum, which includes pathobionts such as *Escherichia coli* and *Klebsiella* [12,99]. These organisms are known to drive inflammation through the production of endotoxins like LPS, which can activate TLRs on immune cells and promote systemic cytokine release. Elevated levels of circulating LPS have been documented in other conditions involving mast cell activation [103] and may similarly play a role in urticaria by priming mast cells and basophils, rendering them more sensitive to non-specific stimuli.

Further supporting this hypothesis, several studies using 16S rRNA gene sequencing to compare the fecal microbiota of CSU patients to healthy controls reported depletion of beneficial bacteria (e.g., *Ruminococcus*, *Lactobacillus*) and enrichment of pro-inflammatory genera (e.g., *Enterobacteriaceae*, *Sutterella*) [89,96,104], which correlated with disease severity scores [105]. These scores are typically based on validated instruments that assess symptoms such as wheal count, itch intensity, and their impact on a patient’s quality of life [1]. Importantly, lower microbial diversity indices (e.g., Shannon, Chao1) were associated with higher Urticaria Activity Scores over 7 days (UAS7) [104,106], indicating a potential relationship between dysbiosis and disease intensity. UAS7 is a validated patient-reported outcome measure used to assess disease activity and control in chronic spontaneous urticaria, based on the sum of daily scores for wheal count and itch intensity over a 7-day period [1]. A significant positive link was observed between the abundance of Bacteroidetes and the presence of symptomatic dermographism. Additionally, Firmicutes abundance positively correlated with both the Urticaria Control Test (UCT) score and basophil FcεRI receptor density, while simultaneously showing a negative correlation with eosinophil count [89]. The ratio between pro- and anti-inflammatory taxa was also positively correlated with CSU severity and refractoriness to antihistamines [104].

Some microbiome alterations also appear to involve metabolic pathways: levels of bacterial metabolites involved in tryptophan metabolism, bile acid conjugation, and SCFA production are altered in CSU [96,107], suggesting a broader disruption of host-microbiome metabolic crosstalk. These findings highlight the possibility that gut-derived immune signals may modulate mast cell behavior not only via cellular priming but also through metabolic reprogramming.

Despite these promising findings, the literature remains preliminary and limited by several methodological challenges, and the results are inconsistent. Most studies to date involve small sample sizes, cross-sectional designs, and heterogeneous patient populations, often lacking standardization in terms of disease phenotype, medication use, diet, and other confounders [90]. Moreover, few investigations include longitudinal data, making it difficult to determine whether microbiome alterations are a cause, consequence, or merely an epiphenomenon of CSU.

Nevertheless, the convergence of data showing reduced microbial diversity, loss of beneficial SCFA-producing taxa, enrichment of *Proteobacteria*, and associations with disease severity scores provides a compelling rationale for further investigation. These early findings suggest that the gut microbiome may play an active role in modulating the immune threshold of urticaria and that future research integrating metagenomics, metabolomics, and clinical phenotyping could lead to the identification of novel biomarkers or therapeutic strategies. Table 3 offers a comparative view of key gut microbiota features observed in patients with CSU compared to healthy controls. However, there are some considerations for interpretation:

- Heterogeneity: findings across studies can be inconsistent due to differences in geographical location, dietary habits, sample size, methodology (e.g., 16S rRNA gene sequencing vs. metagenomics), disease duration, severity, and medication use.
- Causality vs. association: most studies establish associations, not direct causation. Mendelian randomization studies are emerging to explore causal links.
- Individual variation: the “normal” gut microbiome itself has significant inter-individual variation, making it challenging to define a universal “dysbiotic” profile.

In conclusion, while more research with standardized methodologies and larger cohorts is needed, a consistent picture emerges of gut dysbiosis in urticaria patients, characterized by reduced beneficial bacteria, increased opportunistic pathogens, and altered metabolic functions. This supports the growing interest in the gut–skin–immune axis as a key factor in CSU pathogenesis and a potential target for novel therapeutic strategies.

### 4.1. Evidence from Animal Studies: Gut Microbiota Modulation Reduces Mast Cell Hyperreactivity

Animal studies have played a pivotal role in uncovering the immune-regulatory influence of the gut microbiota on mast cells, lending mechanistic support to the hypothesis that dysbiosis may contribute to CSU pathogenesis. Unlike human studies, which often face limitations in controlling for environmental variables, germ-free (GF) and antibiotic-treated animal models allow for highly controlled investigation of microbiome–host interactions, including their effects on immune development and effector cell function.

One of the most consistent findings across studies is that GF mice exhibit dysregulated mast cell responses. One study by Schwarzer et al. showed that germ-free mice exhibited altered functionality of mast cells and their impaired migration into the intestinal and skin tissue, as well as reduced edema formation, allergic diarrhea, and hypothermia after injecting a degranulation-provoking compound, implying altered sensitivity [110]. However, other studies often show hypersensitivity to certain stimuli due to the lack of proper immune programming or a lack of regulatory signals from the microbiota that would normally temper responses. For example, some papers exploring specific food allergy models in germ-free mice describe more severe hypothermia (a sign of anaphylaxis) despite other seemingly “impaired” allergic responses [111,112]. This suggests a complex interplay where the type of stimulus and the context of mast cell activation matter. This hypersensitivity was attributed to enhanced mast cell density and hyperreactivity, suggesting that the microbiota plays a critical role in calibrating mast cell thresholds during immune development.

Recolonization of GF mice with conventional microbiota or administration of specific probiotic strains has been shown to restore immune homeostasis and reduce mast cell hyperreactivity. For example, the study by Kim et al. screened probiotics for their ability to inhibit mast cell degranulation and found *Lactiplantibacillus plantarum HD02* and *MD159* to be effective; in a passive cutaneous anaphylaxis (PCA) model, these strains significantly attenuated vascular permeability caused by mast cell degranulation [113]. *Lactobacillus rhamnosus GG*, *Lactobacillus acidophilus*, and *Lactobacillus paracasei KBL382* were also shown to reduce symptoms of atopic dermatitis in mice [114,115]. These probiotics decreased mast cell infiltration in the skin and suppressed serum IgE levels, suggesting a reduction in mast cell activation.

Mechanistically, these probiotic effects appear to be mediated through multiple, interconnected pathways:

- Enhanced production of SCFAs such as butyrate and acetate, which stabilize mast cells and promote Treg differentiation [116]
- Suppression of pro-inflammatory cytokines like IL-6, IL-17, and TNF [117]
- Inhibition of TLR signaling, which is critical for microbial sensing by immune cells, including mast cells [118]
- Modulation of gut epithelial barrier function, thereby limiting systemic exposure to microbial antigens and endotoxins [119].

In contrast, antibiotic-treated or dysbiotic mice often show increased mast cell activity, elevated serum cytokine levels, and greater intestinal permeability [120,121]. In one study, vancomycin-treated mice developed increased mast cell infiltration and exaggerated skin inflammatory responses when exposed to irritants [122], further highlighting the protective role of a balanced microbiota in maintaining mast cell quiescence. In addition, antibiotic-induced dysbiosis has been shown to reduce colonic Treg populations and increase systemic LPS levels [123], which could lower mast cell activation thresholds.

Although CSU-specific animal models are limited, the conserved immunologic pathways across allergic and inflammatory models (e.g., mast cell regulation via SCFAs, microbial metabolites, and Treg/Th17 balance) strongly suggest that similar mechanisms may be at play in urticaria. Future animal research focused specifically on CSU-like phenotypes and gut–skin immune crosstalk will be invaluable in confirming these links.

Taken together, animal studies provide compelling evidence that the gut microbiota is a central regulator of mast cell behavior and that dysbiosis can tip the immune system toward a pro-inflammatory, mast cell-hyperreactive state. These findings strongly support ongoing exploration of microbiome-based interventions in CSU.

### 4.2. Impact of Probiotics, Prebiotics, Fecal Microbiota Transplantation and Diet in CSU

Given the growing recognition of gut dysbiosis as a potential contributor to CSU, several investigators have explored whether modulating the gut microbiome through probiotics, prebiotics, or dietary interventions might improve disease control. Although this research is still developing, the concept is biologically plausible and supported by preliminary data.

Probiotics, defined as live microorganisms that confer health benefits when administered in adequate amounts, have been the most studied. Clinical trials have primarily investigated strains of *Lactobacillus* and *Bifidobacterium*, known for their anti-inflammatory effects, ability to restore gut barrier function, and support of regulatory T cell responses [124,125,126]. The study by Nettis et al. [124] evaluated the efficacy and safety of a combination of *Lactobacillus salivarius LS01* and *Bifidobacterium breve BR03* in patients with CSU who remained symptomatic despite H1-antihistamine therapy; while a majority did not show significant improvement, a subset did experience mild to complete remission of symptoms, suggesting potential benefit in some individuals. The randomized clinical trial by Dabaghzadeh et al. [125] investigated the effect of a probiotic capsule (containing *Lactobacillus rhamnosus*, *L. casei*, *L. acidophilus*, *L. bulgaricus*, *Bifidobacterium longum*, *B. breve*, and *Streptococcus thermophilus*) as an adjunct therapy to antihistamines in patients with chronic urticaria; the study reported a significant improvement in urticaria activity (UAS7 scores) in the probiotic group compared to the placebo group. Another randomized placebo-controlled trial in children with chronic urticaria used a combination probiotic product (Yimingjia^®^) containing several *Lactobacillus* strains (*L. gasseri LK001*, *L. salivarius LK002*, *L. johnsonii LK003*, *L. paracasei LK004*, *L. reuteri LK005*) and a *Bifidobacterium* strain (*B. breve*); the study found that adjunct therapy with this probiotic improved wheal size and attack frequency at 4 weeks compared to placebo [126].

However, not all trials have produced consistent results. A 2022 blinded randomized controlled trial (RCT) involving a synbiotic containing several *Lactobacillus* strains, *Bifidobacterium longum*, *Streptococcus thermophilus,* and fructo-oligosaccharides (FOS) failed to show significant differences in symptom control between groups [127].

Another limitation across studies is small sample size, variable diagnostic criteria for CSU, and short follow-up duration. A 2023 systematic review and meta-analysis of nine small clinical trials concluded that while probiotic supplementation may offer modest symptom relief, the overall quality of evidence remains low, and larger, well-designed RCTs are needed to establish clinical efficacy [128].

In addition to probiotics, interest has emerged in prebiotics, which are non-digestible dietary components that selectively promote the growth of beneficial gut bacteria. Compounds such as inulin, FOS, and galacto-oligosaccharides (GOS) have been shown in other inflammatory conditions (e.g., inflammatory bowel diseases, eczema) to increase levels of SCFA-producing bacteria and enhance epithelial integrity [129,130,131,132]. Although specific studies on prebiotics in CSU are less common in the current literature, their mechanism of action (i.e., increasing butyrate levels and Treg responses) is relevant to urticaria pathogenesis and warrants further research. However, it is important to note that some research even suggests that certain prebiotics, such as GOS, can induce IgE-mediated allergic reactions in susceptible individuals [133]. Therefore, while promising, more dedicated research is needed to fully delineate the role and specific benefits of prebiotics in urticaria management.

Besides probiotics and prebiotics, the emerging field of postbiotics also holds therapeutic promise for CSU. Postbiotics are non-viable microbial cells and their metabolic byproducts, such as short-chain fatty acids (SCFAs), organic acids, and bacteriocins [134]. Unlike live probiotics, their non-viability may offer a safer, more stable alternative, especially in immune-compromised individuals. These compounds can exert immunomodulatory, anti-inflammatory, and antioxidant effects [135]. Specifically, in the context of inflammatory skin diseases, postbiotics have been shown to help strengthen the epithelial barrier, reduce skin inflammation by downregulating pro-inflammatory cytokines, and improve overall skin health [129,136,137,138]. While research in CSU is still nascent, the mechanisms by which postbiotics act on the gut–skin axis suggest a potential role in modulating the dysregulated immune responses and inflammation characteristic of the disease.

Fecal microbiota transplantation (FMT) is emerging as a novel and intriguing therapeutic avenue for chronic urticaria, particularly in cases refractory to conventional treatments. The rationale for FMT in chronic urticaria stems from the hypothesis that restoring a healthy, diverse, and functionally balanced gut microbiota can ameliorate disease pathogenesis by correcting dysbiosis, enhancing intestinal barrier integrity, and modulating systemic immune responses that drive mast cell activation [90,93]. While the evidence remains preliminary, consisting primarily of small pilot studies and case reports, some suggest positive outcomes, including symptom reduction and even remission in a subset of patients with severe, antihistamine-resistant chronic spontaneous urticaria [139]. However, larger, well-designed randomized controlled trials are critically needed to establish the definitive efficacy, long-term safety, optimal donor selection, and appropriate patient stratification for FMT in chronic urticaria, given the inherent complexities and potential risks associated with the procedure. Its mention in the context of CSU should therefore be cautious and conceptual, rather than as a near-term therapeutic reality.

Dietary interventions also offer a non-pharmacologic strategy to modulate the microbiome. Western dietary patterns, rich in saturated fats, simple sugars, and ultra-processed foods, are associated with dysbiosis and systemic inflammation [140,141,142]. In contrast, the Mediterranean diet, characterized by high intake of fiber, polyphenols, omega-3 fatty acids, and fermented foods, has been associated with increased microbial diversity and reduced inflammatory markers in several immune-mediated diseases [143,144,145,146]. While no large controlled trials have yet assessed dietary interventions in CSU, observational reports suggest that patients who adopt anti-inflammatory diets may experience fewer flares and reduced medication dependence [147,148,149]. Some studies have also reported improvement in CSU control with low-histamine [150,151] or pseudoallergen-free diets [152], although these approaches remain controversial and lack rigorous validation.

Taken together, the available evidence suggests that modulation of the gut microbiome through probiotics, prebiotics, or targeted dietary strategies may represent a valuable adjunct to conventional pharmacotherapy in CSU. While these approaches are unlikely to replace antihistamines or biologics in moderate-to-severe cases, they may offer benefit in specific phenotypes, particularly in patients with mild disease, concomitant gut-related symptoms, or interest in integrative care. Importantly, larger placebo-controlled trials with standardized protocols are needed to confirm efficacy, determine optimal strains and dosages, and identify the patient subgroups most likely to respond. Table 4 summarizes the main microbiome-modulating strategies in CSU.

## 5. Challenges and Future Directions in Microbiome-Targeted Interventions

Despite growing interest in microbiome modulation as a therapeutic strategy for CSU, several important challenges limit the clinical translation of current findings. One of the most critical issues is strain specificity. The effects of probiotics are highly dependent on the exact microbial strain used, as different strains within the same species can exhibit vastly different immunological properties. For example, while some strains of *Lactobacillus rhamnosus* exhibit anti-inflammatory effects and support regulatory T cell responses [153,154], others may have minimal or no impact on host immunity [155]. This makes generalization across studies difficult and underscores the need for precise identification and validation of probiotic strains with reproducible immunomodulatory activity in CSU-relevant models.

Another significant scientific and methodological issue is the lack of established causality; it is currently unclear whether microbial alterations are a cause, a consequence, or merely an epiphenomenon of chronic inflammation in CSU. Most existing studies are cross-sectional and observational in design, limiting the ability to infer directionality or mechanisms of effect [90]. To better understand the dynamic relationship between the microbiome and CSU, longitudinal studies are essential. These should track microbial composition and function over time, alongside clinical disease activity and treatment response, to identify predictive or prognostic microbial signatures. By analyzing microbial composition, diversity, and metabolite production in CSU patients, clinicians may be able to identify distinct microbiota-associated phenotypes or endotypes of disease. Such stratification could help predict disease severity, identify likely responders to microbiome-targeted therapies, and tailor treatment accordingly—a hallmark of personalized medicine. Such studies could also shed light on whether specific microbial patterns precede disease onset, relapse, or remission.

Another major limitation is the absence of large RCTs specifically focused on microbiome-targeted therapies in CSU, together with a lack of standardization in probiotic dosage and treatment duration. Clinical trials investigating probiotics in CSU have employed varying regimens ranging from a few weeks to several months, with inconsistent dosing and strain selection strategies, sample size, outcome measures, and endpoints [90,93]. It remains unclear what constitutes an adequate “dose” for clinical efficacy, whether higher colony-forming unit (CFU) counts are more effective, or how long supplementation needs to be maintained to achieve lasting immunologic or clinical benefit. Furthermore, interindividual differences in baseline microbiota composition may influence probiotic engraftment and function, making personalized approaches increasingly important. As a result, the evidence base remains fragmented and insufficient to support routine clinical use. There is an urgent need for well-powered, placebo-controlled RCTs that evaluate microbiome interventions using standardized protocols, validated clinical endpoints (e.g., UAS7, quality of life), and stratification by urticaria phenotype.

Finally, although high-fiber, anti-inflammatory dietary patterns, such as the Mediterranean diet, are known to support microbial diversity and enhance the abundance of SCFA-producing bacteria [146], their role in CSU has not been directly studied. Nevertheless, these diets are associated with reduced systemic inflammation in other immune-mediated diseases and may represent an indirect but sustainable method to promote microbiome health. However, adherence, cultural dietary habits, and long-term feasibility must be considered when recommending broad dietary changes. More controlled dietary intervention studies are needed to confirm whether such strategies can influence urticaria activity or treatment response.

In parallel, the field would benefit from multi-omics approaches, integrating metagenomics, metabolomics, transcriptomics, and immunophenotyping. This systems-level strategy could help unravel functional pathways linking microbial activity to immune modulation, mast cell regulation, and barrier integrity. The integration of multi-omics technologies, artificial intelligence-driven microbiome analytics, and real-world clinical data may enable the development of predictive models to guide individualized care. For instance, identifying shifts in microbial metabolic products (e.g., SCFAs, bile acids, tryptophan derivatives) alongside cytokine profiling and immune cell phenotyping could offer a more holistic view of the gut–skin–immune axis in CSU. As the field matures, it is conceivable that microbiome-based diagnostics and interventions will form part of a precision immunodermatology toolkit, offering CSU patients more targeted, effective, and sustainable treatment options.

Until such comprehensive studies are conducted, the relationship between the microbiome and urticaria must be regarded as hypothesis-generating rather than clinically actionable. Nonetheless, the convergence of early evidence justifies deeper investigation and investment in microbiome-focused research as a promising avenue for future therapeutic innovation.

**Figure 3:**
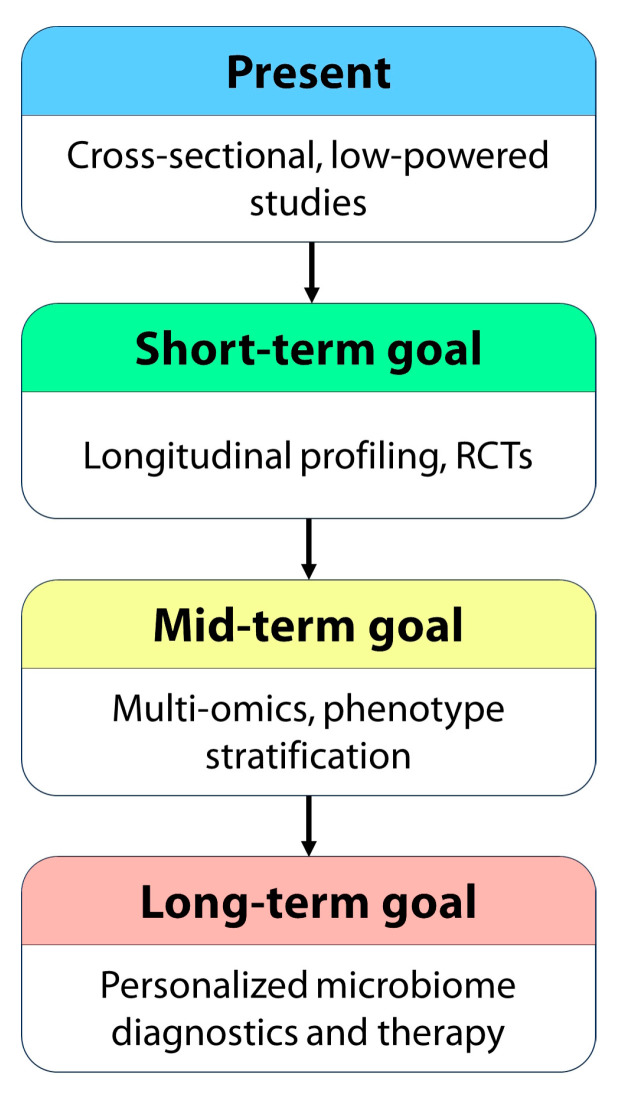
Proposed longitudinal roadmap for advancing microbiome research in CSU. Current research is largely limited to small, cross-sectional studies showing associations between dysbiosis and CSU. Short-term goals include the development of longitudinal cohort studies and small-scale randomized controlled trials (RCTs) evaluating microbiota-targeted interventions. Mid-term objectives involve the integration of multi-omics approaches (e.g., metagenomics, metabolomics, immunophenotyping) and the stratification of CSU phenotypes based on microbiome and immune signatures. The long-term vision includes the implementation of personalized therapeutic strategies informed by microbiome profiling and the development of diagnostic tools to guide treatment decisions.

Figure 3 proposes a longitudinal roadmap for advancing microbiome research in CSU, with short-, mid-, and long-term goals.

## 6. Conclusions

The interplay between the gut microbiome and systemic immune regulation, often referred to as the gut–skin axis, represents an exciting and rapidly evolving frontier in immunodermatology. Although research in CSU is still in its early stages, emerging evidence suggests that gut dysbiosis may contribute to disease pathogenesis by promoting low-grade systemic inflammation, disrupting immune tolerance, and lowering mast cell activation thresholds.

These insights open the possibility that the gut microbiome could serve as both a biomarker and a therapeutic target in CSU. Interventions such as probiotics, prebiotics, dietary strategies, and potentially even more advanced tools like microbiome profiling or fecal microbiota transplantation (FMT) may complement existing therapies and help personalize treatment.

However, before these concepts can be translated into clinical practice, robust mechanistic studies and well-designed clinical trials are essential. Clarifying causality, identifying responsive patient subgroups, and validating intervention strategies will be critical steps in moving from hypothesis to application. With continued interdisciplinary research, the microbiome may offer a novel lens through which we understand and manage urticaria in the years to come.

## Figures and Tables

**Table 1:** Normal human gut microbiome: phyla, genera, and representative species [31,34,35,36]. Dominant phyla in healthy adults are usually Firmicutes and Bacteroidetes. Bifidobacteria (Actinobacteria) are particularly abundant in infants and those consuming high-fiber diets. Proteobacteria are present in low numbers under normal conditions but increase during dysbiosis. Species diversity and abundance vary widely between individuals depending on diet, age, geography, and lifestyle.

Phylum	Representative Genera	Notable Species/Examples	Functions/Notes
Firmicutes	*Faecalibacterium*	*F. prausnitzii*	Anti-inflammatory, SCFA (butyrate) producer
	*Clostridium*	*C. leptum*, *C. coccoides*	SCFA production, immune modulation
	*Lactobacillus*	*L. rhamnosus*, *L. acidophilus*	Probiotic, lactic acid production
	*Ruminococcus*	*R. bromii*, *R. flavefaciens*	Resistant starch degradation
	*Blautia*	*B. obeum*, *B. wexlerae*	SCFA producer, potential metabolic benefits
	*Eubacterium*	*E. rectale*	Butyrate production, colonic health
Bacteroidetes	*Bacteroides*	*B. fragilis*, *B. thetaiotaomicron*, *B. vulgatus*	Carbohydrate metabolism, immune homeostasis
	*Prevotella*	*P. copri*	Fiber fermentation, controversial inflammatory links
	*Alistipes*	*A. putredinis*	Potentially protective, involved in amino acid metabolism
Actinobacteria	*Bifidobacterium*	*B. longum*, *B. breve*, *B. adolescentis*	Early colonizer, probiotic, carbohydrate metabolism
	*Collinsella*	*C. aerofaciens*	Role in lipid metabolism
Proteobacteria	*Escherichia*	*E. coli* (commensal strains)	Vitamin K production, immune interaction (can become pathogenic if dysregulated)
	*Klebsiella*	*K. pneumoniae* (commensal strains)	May act as pathobiont if overgrown
	*Enterobacter*	*E. cloacae*	Often transient, opportunistic potential
Verrucomicrobia	*Akkermansia*	*A. muciniphila*	Mucus layer degradation, metabolic health benefits
Fusobacteria	*Fusobacterium*	*F. nucleatum* (low abundance)	Normally low in abundance; associated with disease in overgrowth
Synergistetes	*Synergistes*	*S. jonesii*	Present in low abundance; limited known function
Tenericutes	*Mycoplasma*	*M. hominis* (rarely detected)	Occasional, typically not dominant in healthy microbiome

**Table 2:** Comparative summary of gut microbiota alterations observed in atopic dermatitis (AD), psoriasis, rosacea, and acne vulgaris. All four conditions exhibit reduced microbial diversity and shifts toward pro-inflammatory bacterial profiles, with associated changes in gut barrier function and systemic immune activation. While evidence is more established in AD and psoriasis, studies investigating the gut microbiome in acne and rosacea remain preliminary. ↓ = decrease; ↑ = increase; SCFA = short-chain fatty acids; FMT = fecal microbiota transplantation.

Feature	Atopic Dermatitis (AD) [62,63,87]	Psoriasis [70,71]	Rosacea [75,77]	Acne Vulgaris [42,82]
Microbial diversity	Decreased	Decreased	Decreased	Decreased
Key changes in composition	↓ *Bifidobacterium*, ↓ *Lactobacillus,* ↑ *Clostridium* clusters (pro-inflammatory)	↓ SCFA producers (*Faecalibacterium prausnitzii*—key anti-inflammatory bacterium), ↓ Actinobacteria, ↑ *Escherichia coli*, ↑ *Ruminococcus gnavus*	↑ *Helicobacter pylori*, ↑ Firmicutes/Bacteroidetes ratio	↑ Firmicutes/Bacteroidetes ratio Shift toward pro-inflammatory taxa
Gut barrier function	Increased permeability (“leaky gut”)	Increased permeability (“leaky gut”)	Increased permeability (“leaky gut”)	Possible increased permeability (less studied)
Associated immune changes	Th2 skewing, elevated IgE, systemic inflammation	Th17 polarization, systemic inflammation	Systemic inflammation	Systemic inflammation, possible hyperandrogenism links
Clinical/Dietary modulators	Probiotics show potential benefits (strain-specific) Breastfeeding protective	Preliminary data on FMT; SCFA-focused dietary interventions under investigation	Probiotics under investigation; dietary modifications (e.g., low-histamine, gluten-free) anecdotally helpful but limited evidence	High-glycemic-load diets linked to gut dysbiosis; probiotics under early investigation
Evidence level	Moderate (RCTs in prevention; smaller studies in treatment)	Moderate (observational studies; pilot interventions)	Preliminary (small studies; emerging field)	Preliminary (small studies; emerging field)

**Table 3:** Comparative overview of gut microbiota features observed in patients with chronic spontaneous urticaria (CSU) versus healthy controls. These alterations support the hypothesis of dysbiosis-driven immune dysregulation in urticaria.

Feature/Genera Category	Observation in Urticaria Patients (Compared to Healthy Controls)	Key Genera/Phyla Examples	Important Notes & Implications
Overall diversity	Alpha diversity: usually decreased or no significant difference [90,108].	-	Alpha diversity measures richness and evenness within a sample. A decrease suggests a less diverse and potentially less resilient microbial community. Some studies report no significant difference, highlighting the need for larger and more standardized cohorts.
	Beta diversity: usually significantly different [63,90,93].	-	Beta diversity measures the differences in microbial composition between groups (e.g., CSU vs. healthy controls). A significant difference indicates distinct microbial communities in urticaria patients.
Genera (Decreased)	Beneficial/Commensal bacteria: often decreased [93,102].	*Lactobacillus* spp. *Bifidobacterium* spp. *Faecalibacterium prausnitzii* *Roseburia* spp. *Bacteroides* spp. (though some studies vary) *Lachnospiraceae family* (many SCFA producers) *Prevotella* spp. (often varied)	These genera are known for producing beneficial metabolites like SCFAs (e.g., butyrate), which are crucial for gut barrier integrity, immune regulation, and anti-inflammatory effects. Their reduction can contribute to increased gut permeability and systemic inflammation.
Genera (Increased)	Opportunistic pathogens/Pro-inflammatory bacteria: often increased [93,105].	*Proteobacteria* phylum, *Enterobacteriaceae* family (*Escherichia coli* and *Klebsiella* spp.) *Peptostreptococcaceae* family (*Clostridioides difficile* and other anaerobes)	An increase in these taxa is often associated with dysbiosis and a pro-inflammatory gut environment. Proteobacteria is often considered a hallmark of dysbiosis and may contribute to increased gut permeability and LPS production.
Phyla level	Firmicutes and Bacteroidetes: often decreased in relative abundance, or altered ratios [89,90].	Firmicutes, Bacteroidetes	These are the two most dominant phyla. While general trends suggest a decrease in ““beneficial” Firmicutes members and some Bacteroidetes, the exact alterations can vary between studies. The Firmicutes-to-Bacteroidetes ratio is often examined, but findings are not always consistent.
	Proteobacteria: often increased [90,92,93].	*Proteobacteria*	An enrichment of Proteobacteria is frequently observed and is often considered a key indicator of gut dysbiosis in various inflammatory conditions.
Functional alteration	Reduced SCFA production: linked to decreased beneficial bacteria [93,105].	-	Dysbiosis can lead to a reduction in SCFA-producing bacteria, which are vital for gut health and immune modulation, potentially exacerbating inflammation in CSU.
	Altered amino acid and bile acid metabolism: due to shifts in microbial communities [109].	-	Changes in the gut microbiota can significantly impact host metabolism, including the processing of amino acids (e.g., tryptophan pathways) and bile acids, which can have systemic immune effects.

**Table 4:** Clinical applications of microbiome-modulating strategies in chronic spontaneous urticaria (CSU). This table summarizes current evidence, known limitations, and the potential role of probiotics, prebiotics, fecal microbiota transplantation (FMT), and dietary interventions, as adjunctive or future therapies. While probiotics show early promise, other strategies remain largely theoretical or investigational and require validation through controlled clinical trials.

Strategy	Current Evidence	Limitations	Potential Role
Probiotics	Preliminary	Strain specificity, RCT gaps	Adjunct to antihistamines and omalizumab
Prebiotics	Theoretical	No CSU trials yet	Microbial support
Fecal microbiota transplant (FMT)	Conceptual	Ethical/regulatory concerns	Future therapy for refractory CSU
Anti-inflammatory diet	Indirect	Low adherence in some patients	Lifestyle adjunct

## Abbreviations

The following abbreviations are used in this manuscript:

AD	Atopic dermatitis
CFU	Colony-forming unit
CIndU	Chronic inducible urticaria
CRP	C-reactive protein
CSU	Chronic spontaneous urticaria
FMT	Fecal microbiota transplantation
FOS	Fructo-oligosaccharides
GF	Germ-free
GOS	Galacto-oligosaccharides
IgE	Immunoglobulin E
IL	Interleukin
LPS	Lipopolysaccharides
PAMPs	Pathogen-associated molecular patterns
RCT	Randomized controlled trial
SCFAs	Short-chain fatty acids
Th	T helper
TLRs	Toll-like receptors
TNF	Tumor necrosis factor
Treg	Regulatory T cell
UAS7	Urticaria Activity Score over 7 days
UCT	Urticaria Control Test
